# Predicting process design spaces for spray drying amorphous solid dispersions

**DOI:** 10.1016/j.ijpx.2021.100072

**Published:** 2021-02-25

**Authors:** Stefanie Dohrn, Pranay Rawal, Christian Luebbert, Kristin Lehmkemper, Samuel O. Kyeremateng, Matthias Degenhardt, Gabriele Sadowski

**Affiliations:** a TU Dortmund University, Department of Biochemical and Chemical Engineering, Laboratory of Thermodynamics, Emil-Figge-Str. 70, D-44227 Dortmund, Germany; b AbbVie Deutschland GmbH & Co. KG, Global Pharmaceutical R&D, Knollstraße, D-67061 Ludwigshafen am Rhein, Germany

**Keywords:** Amorphous solid dispersion, Residual solvent, Spray drying, Solvent selection, PC-SAFT, Process design space, Crystallization, Glass transition

## Abstract

Amorphous solid dispersions (ASDs) are commonly manufactured using spray-drying processes. The product quality can be decisively influenced by the choice of process parameters. Following the quality-by-design approach, the identification of the spray-drying process design space is thus an integral task in drug product development. Aiming a solvent-free and homogeneous ASD, API crystallization and amorphous phase separation needs to be avoided during drying. This publication provides a predictive approach for determining spray-drying process conditions via considering thermodynamic driving forces for solvent drying as well as ASD-specific API/polymer/solvent interactions and glass transitions. The ternary API/polymer/solvent phase behavior was calculated using the Perturbed-Chain Statistical Associating Theory (PC-SAFT) and combined with mass and energy balances to find appropriate spray-drying conditions. A process design space was identified for the ASDs of ritonavir and naproxen with either poly(vinylpyrrolidone) or poly(vinylpyrrolidone-co-vinylacetate) spray dried from the solvents acetone, dichloromethane, or ethanol.

## Graphical abstract

**Figure:**
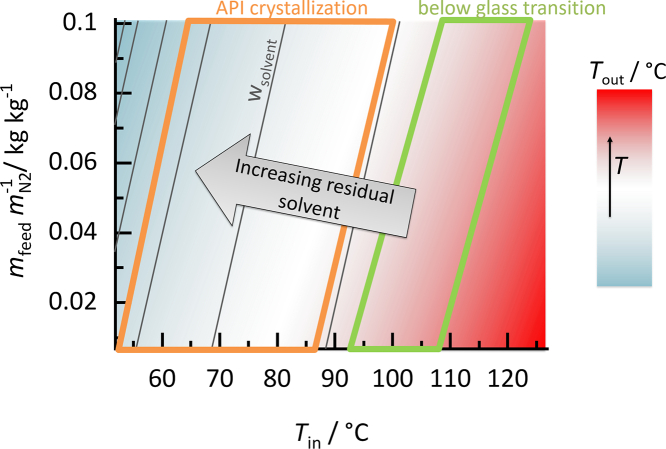

## 1 Introduction

Amorphous solid dispersions (ASDs) increase the bioavailability of poorly-water-soluble active pharmaceutical ingredients (APIs) by embedding the amorphous API in a polymer matrix. (Paudel et al., 2013; Six et al., 2004; Leuner and Dressmann, 2000; Vo et al., 2013) Spray drying is a commonly-used unit operation for the preparation of ASDs.(Dobry Dan et al., 2009; Broadhead et al., 2008; Ziaee et al., 2017; Newman, 2015; van den Mooter, 2012) API and polymer are first dissolved in a solvent or solvent mixture. This mixture (feed solution) is fed into the spray dryer as atomized fine droplets and the solvent is subsequently evaporated. The best-achievable product quality is determined by the macroscopic process-related conditions (e.g. drying temperature and solvent content in the drying gas) and microscopic/intermolecular properties, i.e. the thermodynamic phase behavior of the API/polymer/solvent mixture. Besides unwanted liquid-liquid (amorphous) phase separation and API crystallization, residual solvent can negatively influence the kinetic stability of an ASD, as residual solvent leads to an enhanced molecular mobility, and thus to kinetic destabilization.(Dohrn et al., 2021; Luebbert et al., 2018a; Prudic et al., 2014a; Prudic et al., 2015)

Many researchers have investigated the phase behavior of API/polymer systems for predicting the long-term stability of ASDs.(Prudic et al., 2014a; Burnett et al., 2006; Kyeremateng et al., 2014; Lehmkemper et al., 2017a; Lehmkemper et al., 2017b) Solvent-induced phase separation and unwanted API crystallization were successfully described using thermodynamic models like Flory-Huggins(Duarte et al., 2015) and the Perturbed-Chain Statistical Associating Fluid Theory (PC-SAFT).(Luebbert et al., 2018b; Gross and Sadowski, 2001) Although the solvent is not present in the final ASD anymore after drying, it might affect the ASD during the manufacturing processes and therewith also the final product quality, e.g. by affecting API solubility and glass transition (*T*_g_), or by inducing liquid-liquid phase separation.(Dohrn et al., 2021; Luebbert et al., 2018a) Several solvent effects on the ASD stability or homogeneity are reported in literature(Costa et al., 2016; Wu et al., 2011; Rashid et al., 2014; Wan et al., 2013) and discussed regarding solubility, solvent-induced phase separation, residual-solvent content, and *T*_g_ using ternary phase diagrams of API/polymer/solvent systems. (Dohrn et al., 2021; Luebbert et al., 2018a; Dohrn et al., 2020a; Dohrn et al., 2020b)

The manufacturing of ASDs via spray drying is widely reported in literature. (Newman, 2015; Duarte et al., 2015; Shah et al., 2014; Kutz and Wolff, 2007; Ende et al., 2019; Singh and van den Mooter, 2016) Among others, iterative design of experiments (Maltesen et al., 2008) and process-development flowchart methodologies (Dobry Dan et al., 2009) for spray dryers containing thermodynamic and droplet-drying-kinetic models have been applied for designing the industrial manufacturing of ASDs. (Vehring, 2008) Accounting for the thermodynamics in spray-dryer models usually means including vapor-liquid equilibrium and crystalline API solubility for preparing the initial feed solution prior to the drying process. However, to obtain a homogeneous ASD via spray drying, the phase behavior (phase separation and API crystallization) of API/polymer/solvent systems must be considered as a critical quality attribute throughout the whole spray-drying process. (Dohrn et al., 2021; Luebbert et al., 2018a)

**Fig. 1:**
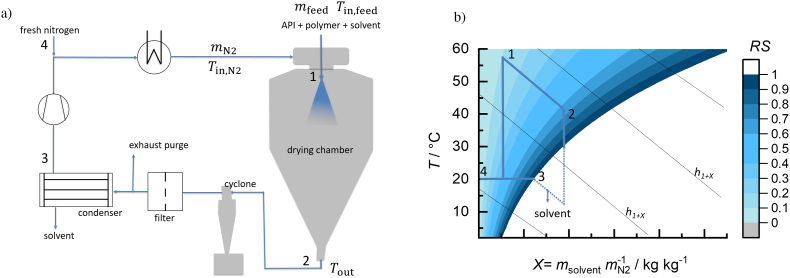
Schematic process flow diagram of the spray dryer (a) and drying process illustrated in a *h-X* diagram for a solvent/nitrogen mixture at *p* = 1.013 bar (b).

Drying processes are often designed using so-called *h-X* diagrams, (Kutz and Wolff, 2007; Baehr and Kabelac, 2006) developed by Richard Mollier for water. (Bauer et al., 1999) The ASD spray-drying process with nitrogen and a solvent cycle is illustrated in a process flow diagram in Fig. 1a and in a *h-X* diagram in Fig. 1b (Baehr and Kabelac, 2006). *h-X* diagrams show the relationship between solvent load *X*, relative saturation (*RS*) and temperature *T*. The solvent load *X* (Eq. (1)) is the total mass of solvent in both, vapor and liquid (*m*_solvent_) related to the mass of nitrogen (*m*_N2_). *RS* (Eq. (2)) is the ratio of the solvent partial pressure *p*_solvent_ and the vapor pressure of the pure solvent (*p*_solvent_^LV^). For water, *RS* is called relative humidity (*RH*). *h*_*1+X*_ is defined as the enthalpy (*H*) of the solvent (vapor AND liquid)/nitrogen mixture related to the mass of nitrogen (*m*_N2_) (Eq. (3)). (Baehr and Kabelac, 2006)

(1) $$X=\frac{m_{\text{solvent}}}{m_{N2}}\;$$

(2) $$\mathrm{RS}=\frac{p_{\text{solvent}}\;}{p_{\text{solvent}}^{\mathrm{LV}}}$$

(3) $$h_{1+X}\;\left(T,X\right)=\frac{H}{m_{N2}}=h_{N2}\;\left(T\right)+X\cdot \;h_{\text{solvent}}\;\left(T\right)$$

Spray drying ASDs starts with preparing an API/polymer/solvent feed mixture, where API and polymer are completely dissolved in the solvent. In the next step, the feed is pumped through a nozzle, creating small droplets from which solvent evaporates within milliseconds.(Maltesen et al., 2008) The solvent drying is accomplished by the contact of the droplets with hot low solvent-loaded nitrogen (point 1 in Fig. 1) in the drying chamber. Drying takes place along the spray-drying chamber. Consequently, the nitrogen is increasingly loaded with the evaporating solvent causing a temperature decrease of the nitrogen when reaching the outlet of the spray dryer (point 2 in Fig. 1). The dried ASD powder is collected in a cyclone separator while the solvent-loaded gas might be recycled. By passing through a particle filter and a cooling trap, the solvent is partially condensed at low temperatures (point 3 in Fig. 1). The recycled nitrogen is then mixed with fresh nitrogen (point 4 in Fig. 1), reheated in the process heater, and fed back into the spray-drying chamber. In a secondary drying step, the residual solvent is usually further removed from the powder to fulfil regulatory requirements with respect to residual solvent.(ICH Expert Working Group, 2019) This step is often costly and time-consuming. Thus, choosing appropriate inlet conditions for the spray dryer, at best saving or limiting a secondary drying step, is beneficial. Process parameters like feed-inlet temperatures and drying-gas rates influence the solvent-drying behavior, which in turn influences the resulting outlet temperature and the residual solvent content remaining in the ASD.

Dobry et al. (Dobry Dan et al., 2009; Newman, 2015) presented a plot of an example spray-drying chart also containing resulting outlet temperatures and residual-solvent contents. However, unwanted phase changes in the ASD during drying, API crystallization or glass transitions were not accounted for in these works and have not been considered so far. The thermodynamic phase behavior of API/polymer/solvent mixtures was intensively experimentally validated in previous works. (Dohrn et al., 2021; Luebbert et al., 2018a; Prudic et al., 2015) In this work, reasonable process design spaces avoiding liquid-liquid phase separation during drying were predicted. For that purpose, inlet temperature, API load in the ASD, selected solvent and solvent partial pressure (i.e. nitrogen mass flows) were varied and resulting outlet temperatures and ASD quality attributes, such as residual-solvent contents, glass transitions and possible phase changes during drying were predicted as function of process parameters. The proposed approach considers the microscopic changes (phase changes/glass transitions) in the ASD particles as function of (changing) solvent contents combined with mass and energy balances on the macroscopic scale. As the focus of this work is on determining the thermodynamic limits for the best-achievable product quality, kinetic effects were not considered here. Thus, real spray-drying processes, which are subject to mass transfer limitations, will always lead to worse results.

Process design spaces were predicted for four ASDs containing the APIs naproxen (NAP) or ritonavir (RIT) with the polymers poly(vinylpyrrolidone) (PVP) or poly(vinylpyrrolidone-*co*-vinylacetate) (PVPVA64). Dichloromethane (DCM), acetone and ethanol were considered as solvents. DCM belongs to Class 2 of the ICH (International Council for Harmonisation) guidelines, (ICH Expert Working Group, 2019) while acetone and ethanol are attributed to the less-toxic and therefore belong to Class 3.

## 2 Materials and Methods

### 2.1 Materials

The polymers PVP with a weight-average molar mass of 1220000 g mol^−1^ (Kollidon® K90), and the copolymer PVPVA64 (Kollidon® VA64) with a weight-average molar mass of 65000 g mol^−1^ were purchased from BASF (Ludwigshafen, Germany). Naproxen was purchased from Sigma-Aldrich (Steinheim, Germany), ritonavir was obtained from AbbVie Deutschland GmbH & Co. KG (Ludwigshafen, Germany).

### 2.2 Heat-capacity measurements

Modulated differential scanning calorimetry (mDSC) was used to measure heat capacities of APIs and polymers using the TA Instruments Q2000 device (Eschborn, Germany). The experimental conditions regarding modulation parameters, pan type, purge gas, and calibration procedure were considered as in an earlier work. (Zhou et al., 2002) The temperature was calibrated using indium and the heat capacity was calibrated using sapphire. 10–15 mg of each sample were transferred into standard aluminum pans with a pinhole lids and put into the measurement cell, which was purged with 50 mL/min of nitrogen. The sample was heated from 0 to 250 °C with a modulated heating ramp of 10 K min^−1^ (heating-only procedure; heat amplitude of 2.65 K at a period of 100 s). It was then kept isothermal for 5 min before it was cooled at 10 K min^−1^ to 0 °C and kept isothermal for 5 min. The suitability of the calibration procedure and the experimental conditions were validated by comparing the measurement results with literature data for NAP (Buchholz et al., 2016) and RIT, (Zhou et al., 2002) which were in good agreement. The detailed measurement results are given in the supplement (Fig. S1 and Table S1).

## 3 Describing the macroscopic behavior of a spray dryer

### 3.1 Mass and energy balances

**Fig. 2:**
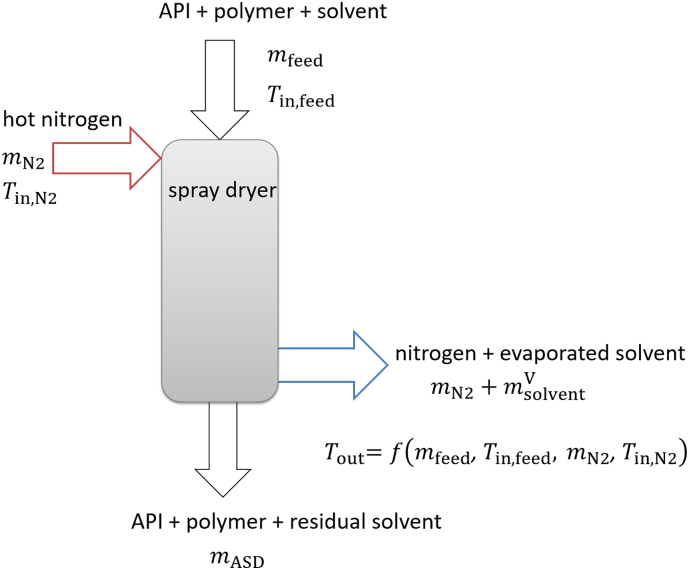
Schematic representation of an adiabatic spray dryer with inlet and outlet mass flows.

The mass flows into and out of a continuously-operated spray dryer are illustrated in Fig. 2. Whereas the input mass flows of the API/polymer/solvent mixture (*m*_feed_) and nitrogen (*m*_N2_) are of different temperatures, the outgoing mass flows (ASD and solvent-loaded nitrogen) were assumed to have the same temperature at steady state. It was further assumed that the spray dryer operates adiabatically, i.e. without heat losses. However, it is also possible to add device-specific heat losses to the calculation, but this was not part of this work.

The energy balance of an adiabatic spray dryer is shown in Eq. (4). It comprises the inlet feed mass (*m*_feed_) consisting of the API and polymer dissolved in the organic solvent, the nitrogen mass m_N2_, the ASD product mass m_ASD_ consisting of API, polymer, and the residual solvent and the mass of solvent which is evaporated (*m*_solvent_^V^).

(4) $$m_{\text{feed}}c_{p,\text{feed}}^{L}\left(T_{\text{in},\text{feed}}-T_{\mathrm{ref}}\right)+m_{N2}c_{p,N2}^{V}\left(T_{\text{in},N2}-T_{\mathrm{ref}}\right)=m_{\mathrm{ASD}}c_{p,\mathrm{ASD}}^{L}\left(T_{\text{out}}-T_{\mathrm{ref}}\right)+m_{N2}c_{p,N2}^{V}\left(T_{\text{out}}-T_{\mathrm{ref}}\right)+m_{\text{solvent}}^{V}\;\left[c_{p,\text{solvent}}^{L}\left(T_{\text{in},\text{feed}}-T_{\text{solvent}}^{\mathrm{LV}}\right)+∆h_{\text{solvent}}^{\mathrm{LV}}\left(T\right)+c_{p,\text{solvent}}^{V}\left(T_{\text{out}}-T_{\mathrm{ref}}\right)\right]$$

*T*_ref_ is the reference temperature, which was set to 273.15 K. *T*_solvent_^LV^ is the solvent boiling temperature at atmospheric pressure. The temperature-dependent solvent evaporation enthalpies ∆*h*_solvent_^LV^ of dichloromethane (DCM), acetone, ethanol, and water were calculated using the approach proposed by Yaws. (Yaws, 1998) The required parameters as well as the critical temperatures *T*^*c*^ are listed in Table 1.

**Table 1:** Solvent evaporation enthalpies used in this work.(Yaws, 1998)

∆*h*_solvent_^LV^ / kJ mol^−1^ = *A* (1-*T/T*^*c*^*)*^*n*^ (*T* in K)			
	*A*	*T*^*c*^	*n*
DCM	41.910	510.00	0.410
Acetone	49.244	508.20	0.481
Ethanol	43.122	516.25	0.079
Water	52.053	647.13	0.321

Molar heat capacities of solvents and nitrogen were considered as being temperature dependent and are listed in Table 2.

**Table 2:** Molar heat capacities at constant pressure of solvents and nitrogen used in this work.(Yaws, 1998)

c_*p*_ / J mol^−1^ K^−1^ = A + B *T+* C *T*^*2*^ *+* D *T*^*3*^ + E *T*^*4*^*(T* in K)						
		A	B	C	D	E
Acetone	V	35.918	9.3896 ·10^−2^	1.8730·10^−4^	−2.1643·10^−7^	6.3174·10^−11^
	L	46.878	6.2653·10^−1^	−2.0761·10^−3^	2.9583·10^−6^	0
DCM	V	26.694	8.3984·10^−2^	8.9712·10^−6^	−5.0924·10^−8^	1.8726·10^−11^
	L	38.941	4.9008·10^−1^	−1.6224·10^−3^	2.3069·10^−6^	0
Ethanol	V	27.091	1.1055·10^−1^	1.0957·10^−4^	−1.5046·10^−7^	4.6601·10^−11^
	L	59.342	3.6358·10^−1^	−1.2165·10^−3^	1.8030·10^−6^	0
Water	V	33.933	−8.4186·10^−3^	2.9906·10^−5^	−1.7825·10^−8^	3.6934·10^−12^
	L	92.053	−3.9953·10^−2^	−2.1103·10^−4^	5.3469·10^−7^	0
Nitrogen	V	29.342	−3.5395·10^−3^	1.0076·10^−5^	−4.3116·10^−9^	2.5935·10^−13^

The temperature-dependent specific heat capacities of APIs and polymers at constant pressure were determined experimentally. The linear interpolation of the experimental values is given in Table 3. Due to the increased molecular mobility, the heat capacity of amorphous materials is notably larger above their glass transition compared to the heat capacity below glass transition. Therefore, two temperature-dependent heat capacities are listed for each, the polymers PVP and PVPVA64, and the APIs RIT and NAP. The amorphous heat capacity below the glass transition was not measurable via DSC for NAP due to rapid recrystallization. For RIT, however, the amorphous heat capacity below glass transition is almost identical to the crystalline heat capacity. Therefore, it can be assumed that the amorphous heat capacity of NAP below glass transition is in the same order as the crystalline heat capacity of NAP. Depending on the state of the components, the respective *c*_*p*_ should be selected, whereby the heat capacities of APIs and polymers have a minor effect on the calculation of the design space.

**Table 3:** Heat capacities of APIs and polymers used in this work.

*c*_*p*_ / J kg^−1^ K^−1^ = A + B *T (T* in K), measured temperature region C ≤ *T* ≤ D					
	*A*	*B*	*C*	*D*	State
NAP	330.509	3.0128	273.15	429.47	crystalline
	1585.386	1.074	429.47	492.15	amorphous, above *T*_*g*_
RIT	311.732	3.306	273.15	398.12	crystalline
	458.502	2.920	273.15	323.50	amorphous, below *T*_*g*_
	1450.001	1.391	323.50	473.15	amorphous, above *T*_*g*_
PVP	901.372	1.914	273.15	446.15	below *T*_*g*_
	1132.134	1.824	446.15	493.15	above *T*_*g*_
PVPVA64	193.256	3.978	273.15	384.15	below *T*_*g*_
	859.895	2.813	446.15	493.15	above *T*_*g*_

There are a number of estimation methods for heat capacities of pure liquids but very few correlations have been suggested for mixtures. (Reid et al., 1977; Teja, 1983) Due to the lack of experimental data, heat capacities of the ternary API/polymer/solvent mixtures were approximated by simple mixing rules applying the mass fractions w of API, polymer, and solvent (Eq.s (5) and (6)). (Reid et al., 1977) This approach to estimating the heat capacities of ASDs is erroneous and can only serve as an imprecise estimate, whereby the effect on the calculation of the design space is likely to be minor.

(5) $$c_{p,\text{feed}}^{L}=w_{\mathrm{API},\text{feed}}c_{p,\mathrm{API}}^{L}+w_{\text{polymer},\text{feed}}c_{p,\text{polymer}}^{L}+w_{\text{solvent},\text{feed}}c_{p,\text{solvent}}^{L}$$

(6) $$c_{p,\mathrm{ASD}}^{L}=w_{\mathrm{API},\mathrm{ASD}}c_{p,\mathrm{API}}^{L}+w_{\text{polymer},\mathrm{ASD}}c_{p,\text{polymer}}^{L}+w_{\text{solvent},\mathrm{ASD}}c_{p,\text{solvent}}^{L}$$

While the solvent partially evaporates from the liquid into the vapor phase, polymer and API remain in the liquid phase. The mass of the (residual-solvent containing) ASD product was therefore calculated as the difference between feed and evaporated solvent (*m*_solvent_^V^) (Eq. (7)).

(7) $$m_{\mathrm{ASD}}=m_{\text{feed}}-m_{\text{solvent}}^{V}$$

The outlet temperature *T*_out_ of the spray dryer can thus be obtained from combining Eq.s (4)–(7) to Eq. (8):

(8) $$T_{\text{out}}=T_{\mathrm{ref}}+\frac{m_{\text{feed}}c_{p,\text{feed}}^{L}\left(T_{\text{in},\text{feed}}-T_{\mathrm{ref}}\right)+m_{N2}c_{p,N2}^{V}\left(T_{\text{in},N2}-T_{\mathrm{ref}}\right)-m_{\text{solvent}}^{V}\;\left[c_{p,\text{solvent}}^{L}\left(T_{\text{in},\text{feed}}-T_{\text{solvent}}^{\mathrm{LV}}\right)+∆h_{\text{solvent}}^{\mathrm{LV}}\left(T\right)\right]}{\left(m_{\text{feed}}-m_{\text{solvent}}^{V}\;\right)c_{p,\mathrm{ASD}}^{L}+m_{N2}c_{p,N2}^{V}+m_{\text{solvent}}^{V}c_{p,\text{solvent}}^{V}}$$

The mass of evaporating solvent *m*_solvent_^V^ was determined by the vapor-liquid equilibrium (VLE), described by Eq. (9).(Luebbert et al., 2018a) Here, *y*_solvent_ is the mole fraction of the solvent in the vapor phase, p is the total pressure (the product of these two is the solvent partial pressure used in Eq. (2)) and *p*_solvent_^LV^ is the solvent vapor pressure which depends on temperature. *γ*_solvent_^L^ is the solvent activity coefficient in the liquid phase (containing API, polymer and solvent). It accounts for the non-idealities in the API/polymer/solvent mixture and was calculated in this work using PC-SAFT(Gross and Sadowski, 2001) (Section 4.3).

(9) $$\mathrm{RS}=\frac{y_{\text{solvent}}\;p\;}{p_{\text{solvent}}^{\mathrm{LV}}}=x_{\text{solvent}}\;\gamma_{\text{solvent}}^{L}$$

The total pressure p is obtained by the sum of the partial pressures of solvent and nitrogen (*p*_solvent_ and *p*_N2_):

(10) $$p=p_{\text{solvent}}+p_{N2}\;$$

By solving the energy balance (Eq. (8)) simultaneously with Eq. (9), the evaporating solvent mass (*m*_solvent_^V^) was calculated as function of the nitrogen inlet temperature and the mass of nitrogen. To identify the process design space for a whole range of drying conditions, the inlet temperature was varied between 50 °C and 130 °C, and the drying ratio (*m*_feed_/*m*_N2_) was varied between 0.01 and 0.08 resulting in a grid of 240 calculation points. The results of the predicted drying behavior (*T*_out_ and solvent content in the ASD at the outlet) were used afterwards for investigating whether the API concentration in the final polymer/solvent mixture (ASD) exceeds the API solubility (Section 4.1, Eq. (12)) and whether the ASD is below or above its glass transition (Section 4.2, Eq. (13)).

### 3.2 Calculation of *h-X* diagrams

*h-X* diagrams of solvent/nitrogen systems were used to visualize the thermodynamic states of the vapor phase during drying. For that purpose, nitrogen was treated as an ideal gas and the vapor pressures of the solvents (see Eq. (2)) were calculated using PC-SAFT.(Gross and Sadowski, 2001) The enthalpy *h*_*1+X*_ was obtained by Eq. (11).(Baehr and Kabelac, 2006)

(11) $$h_{1+X}\;\left(T,X\right)=c_{p,N2}^{V}\;\left(T-T_{\mathrm{ref}}\right)+X\;\left[∆h_{\text{solvent}}^{\mathrm{LV}}\;\left(T\right)+c_{p,\text{solvent}}^{L}\;\left(T-T_{\mathrm{ref}}\right)\right]$$

*c*_*p*, solvent_^L^ and *c*_*p*, solvent_^V^ are the solvent heat capacities in the liquid (L) and the vapor state (V), *c*_*p*, N2_^V^ is the heat capacity of nitrogen (all listed in Table 2). ∆*h*_solvent_^LV^(*T*) is the solvent evaporation enthalpy (listed in Table 1). *T* is the temperature and *T*_ref_ is the reference temperature (273.15 K).

## 4 Describing the microscopic behavior of the ASD during drying

### 4.1 General phase behavior of API/polymer/solvent systems

**Fig. 3:**
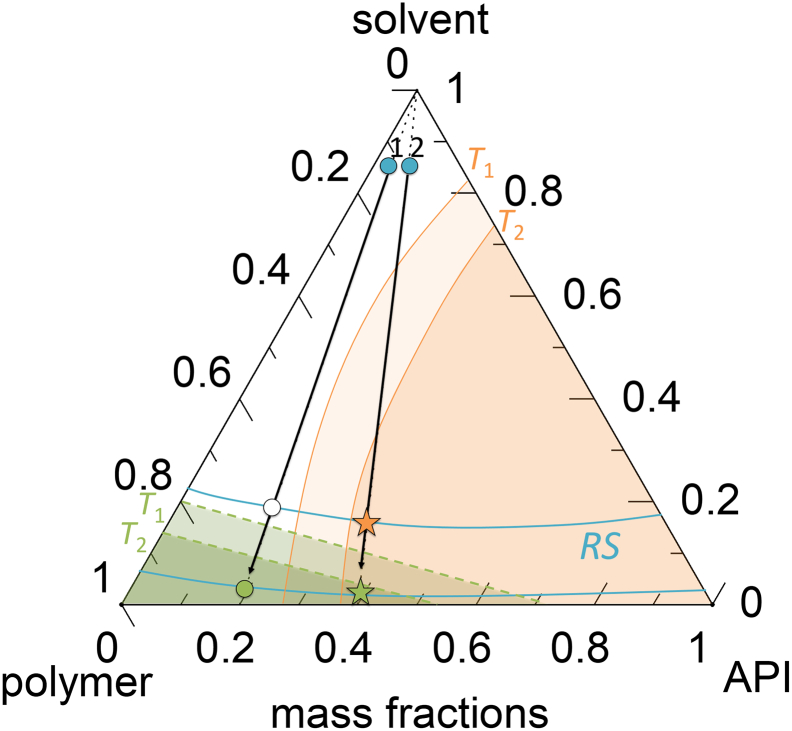
Schematic drying process in a ternary API/polymer/solvent system. The API solubility at different temperatures *T*_1_ and *T*_2_ (*T*_2>_*T*_1_) are indicated by orange lines, API-supersaturated regions are the orange regions. Blue lines indicate the residual-solvent contents at certain solvent *RS*, glass transitions at different system temperatures are shown as green dashed lines. Regions below the glass transition are shown as green regions. The drying process is illustrated for two different initial feeds with API loads of *w*_API_^ASD^ = 0.2 and 0.4 in the solvent-free ASD (blue circles 1 and 2) by black arrows resulting in a thermodynamically-stable ASD above glass transition (white circle), a thermodynamically and kinetically-stable ASD (green circle), a thermodynamically-instable and kinetically-metastable ASD (orange star) or a thermodynamically-instable and kinetically-stabilized ASD below glass transition (green star). (For interpretation of the references to colour in this figure legend, the reader is referred to the web version of this article.)

The ternary phase behavior of an API/polymer/solvent system during drying is schematically presented in Figure 3.(Dohrn et al., 2021; Luebbert et al., 2018a)

The ternary phase diagram includes results of vapor-liquid-equilibrium (VLE) calculations, which determine the residual-solvent content at a certain outlet *RS* and temperature. The solubility lines obtained from solid-liquid-equilibrium (SLE) calculations determine whether an ASD might crystallize or not and the glass transition indicates the transition between glassy and liquid states. Below glass transition, solvent drying is remarkably slowed down and ASDs in glassy regions might remain amorphous for certain time and are thus kinetically stabilized against crystallization even at supersaturated API concentrations.

A drying particle is exposed to different temperatures in a spray dryer. This also needs to be considered when determining suitable process conditions. Higher temperatures lead to higher API solubility as well as to a smaller glassy region. Thus, the crystallization regions and the glassy regions both decrease with temperature (schematically illustrated in Fig. 3 by two temperatures *T*_1_ *<* *T*_2_). (Dohrn et al., 2021)

The drying process starts with an initially-homogeneous liquid feed in the solvent-rich corner of the ternary diagram (two example ASDs with different drug loads are indicated in Fig. 3). Since only the solvent evaporates, drying follows the black arrows at a constant API/polymer ratio. The drying end point is determined by the spray-drying outlet temperature *T*_out_ and the residual solvent content in the ASD. In case 1 (Fig. 3), the API load in the solvent-free ASD is *w*_API_^ASD^ = 0.2. Solvent evaporates from the liquid phase until *T*_out_ is reached. Depending on the drying conditions (inlet temperature and solvent partial pressure), drying may result in thermodynamically-stable ASDs located above (white circle in Fig. 3) or below the glass transition (green circle in Fig. 3). Case 2 in Fig. 3 shows the drying process for a higher API load (*w*_API_^ASD^ = 0.4) in the ASD. During drying, the mixture enters the API-supersaturated region. Therefore, the drying will result in partially-crystallized ASDs above (orange star in Fig. 3) or below glass transition (green star Fig. 3).

### 4.2 Calculation of phase equilibrium and glass transition

The system of API, polymer, and solvent may exhibit (possibly coupled) phase equilibrium between vapor (V), liquid (L), and crystal (S) phases. For the modeling, it was assumed that the vapor phase does neither contain API nor polymer. Solvent vapor might be absorbed by the liquid (amorphous) phase and thus solvent might be present in both, vapor and liquid. The polymers can only be present in the liquid (amorphous) phase - they neither evaporate nor crystallize. API might be either amorphously dissolved in the liquid phase or it might be crystalline (solid phase).

The thermodynamic equilibrium between solvent vapor and the liquid API/polymer/solvent mixture was modeled by Eq. (9) (Section 3.1). To evaluate the risk of API crystallization, the solubility of the crystalline API (*x*_API_^L^) in a solvent, in an amorphous polymer, or in a mixture thereof was calculated at given temperature *T* according to Eq. (12).(Prausnitz et al., 1999)

(12) $$x_{\mathrm{API}}^{L}=\frac{1}{\gamma_{\mathrm{API}}^{L}}\exp\left\{-\frac{{\Delta h}_{\mathrm{API}}^{\mathrm{SL}}}{R\;T}\;\left(1-\frac{T}{T_{\mathrm{API}}^{\mathrm{SL}}}\right)-\frac{{\Delta c}_{p,\mathrm{API}}^{\mathrm{SL}}}{R}\;\left[\ln\left(\frac{T_{\mathrm{API}}^{\mathrm{SL}}}{T}\right)-\frac{T_{\mathrm{API}}^{\mathrm{SL}}}{T}+1\right]\right\}\;$$

*γ*_API_^L^ is the activity coefficient of the API in the liquid phase, *R* is the universal gas constant (8.3145 J mol^−1^ K^−1^), *T*_API_^SL^ is the API melting temperature, Δ*h*_API_^SL^ is the API enthalpy of fusion, and Δ*c*_*p*, API_^SL^ is the difference in the solid and liquid heat capacities of the API. The API melting properties used in this work were taken from literature as summarized in Table 4.

**Table 4:** Melting properties, densities, and glass-transition temperatures of the substances investigated in this work.

	*T*_API_^SL^ / K	Δ*h*_API_^SL^ / kJ mol^−1^	Δ*c*_*p*, API_^SL^ / J mol^−1^ K^−1^	*T*_*g*_ / K	*ρ* / kg m^−3^
NAP	429.47 (Paus, 2015)	31.50 (Paus, 2015)	87.44 (Paus, 2015)	265.15 (Prudic et al., 2015)	1250 (Paudel et al., 2013)
RIT	398.12 (Dohrn et al., 2021)	63.20 (Dohrn et al., 2021)	224.16 (Dohrn et al., 2021)	323.50 (Dohrn et al., 2021)	1151 (Dohrn et al., 2021)
PVP				446.15 (Dohrn et al., 2020a)	1250 (Hancock et al., 1995)
PVPVA64				384.15 (Prudic et al., 2014b)	1190 (Six et al., 2004)
Acetone				142.15 (Hsieh et al., 2014)	790 (Luebbert et al., 2018a)
DCM				103.05 (Lesikar, 1976)	1330 (Luebbert et al., 2018a)
Ethanol				96.15 (Carpenter et al., 1967)	790 (Luebbert et al., 2018a)
Nitrogen					1.1496 (Span et al., 2000)

The glass transition of the spray-dried liquid phase consisting of API, polymer, and solvent was predicted using the Gordon-Taylor equation for ternary systems (Eq. (13)).(Lu and Zografi, 1998)

(13) $$T_{g}=\frac{w_{\text{polymer}}^{L}T_{g,\text{polymer}}+K_{\text{polymer},\mathrm{API}}w_{\mathrm{API}}^{L}T_{g,\mathrm{API}}+K_{\text{polymer},\text{solvent}}w_{\text{solvent}}^{L}T_{g,\text{solvent}}}{w_{\text{polymer}}^{L}+K_{\text{polymer},\mathrm{API}}w_{\mathrm{API}}^{L}+K_{\text{polymer},\text{solvent}}w_{\text{solvent}}^{L}}$$

*w*_polymer_^L^*, w*_API_^L^ and *w*_solvent_^L^ are the mass fractions of polymer, API, and solvent, respectively. *T*_g,polymer_, *T*_g,API_ and *T*_g,solvent_ are the glass-transition temperatures of polymer, API, and solvent. The Gordon-Taylor interaction parameters *K* between polymer/API and polymer/solvent were predicted using Eq. (14).(Lu and Zografi, 1998)

(14) $$K_{\text{polymer},\mathrm{API}}=\frac{\rho_{\text{polymer}}T_{g,\text{polymer}}}{\rho_{\mathrm{API}}T_{g,\mathrm{API}}}\;K_{\text{polymer},\text{solvent}}=\frac{\rho_{\text{polymer}}T_{g,\text{polymer}}}{\rho_{\text{solvent}}T_{g,\text{solvent}}}\;$$

The pure-components' glass-transition temperatures *T*_*g*_ and densities *ρ*_polymer_*, ρ*_API*,*_ *ρ*_solvent_ were taken from literature and are also listed in Table 4.

### 4.3 PC-SAFT

The solvent vapor pressures, as well as the activity coefficients *γ* used for calculating phase equilibrium were obtained from the thermodynamic model PC-SAFT.(Gross and Sadowski, 2001) PC-SAFT determines the residual Helmholtz energy *a*^*res*^ via summing up specific molecular contributions caused by repulsion (hard chain *a*^*hc*^), attraction (dispersion *a*^*disp*^) and association (*a*^*assoc*^) according to Eq. (15).(Gross and Sadowski, 2002)

(15) $$a^{\mathrm{res}}=a^{\mathrm{hc}}+a^{\mathrm{disp}}+a^{\mathrm{assoc}}$$

Each molecule has a defined number of segments (*m*_*i*_^*seg*^*)* with segment diameter *σ*_*i*_ and a dispersion energy parameter (*u*_*i*_/*k*_*B*_). Hydrogen-bond forming molecules are additionally characterized by the number of association sites *N*_*i*_^*assoc*^, the association energy (*ε*^*A*_*i*_*B*_*i*_^/*k*_*B*_), and the association volume *κ*^*A*_*i*_*B*_*i*_^. The pure-component parameters of the substances investigated in this work are summarized in Table 5. In mixtures, conventional Berthelot(Berthelot and Gaz, 1898)-Lorentz(Lorentz, 1881) combining rules were used to obtain the segment diameter *σ*_*ij*_ (Eq. (16)) and the dispersion energy *u*_*ij*_ (Eq. (17)).

(16) $$\sigma_{\mathrm{ij}}=\frac{1}{2}\cdot \left(\sigma_{i}+\sigma_{j}\right)$$

(17) $$u_{\mathrm{ij}}=\left(1-k_{\mathrm{ij}}\right)\sqrt{u_{i}\;u_{j}}$$

**Table 5:** PC-SAFT pure-component parameters used in this work.

	*M /*g mol^−1^	*m*_*i*_^*seg*^*M*^*−1*^*/*mol g^−1^	*σ*_*ι*_*/*Å	*u*_*i*_*k*_*B*_^*−1*^*/*K	*ε*^*A*_*i*_*B*_*i*_^*k*_*B*_^*−1*^*/*K	*κ*^*A*_*i*_*B*_*i*_^	*N*^*assoc*^
PVP (Prudic et al., 2014b)	1220000	0.0407	2.710	205.60	0	0.02	10977/10977
PVPVA64 (Lehmkemper et al., 2017b)	65000	0.0372	2.947	205.27	0	0.02	653/653
NAP (Prudic et al., 2014b)	230.26	0.0352	2.939	229.45	934.2	0.02	2/2
RIT(Dohrn et al., 2021)	721.00	0.0220	3.900	305.79	1040.9	0.02	4/4
acetone (Tumakaka and Sadowski, 2004)	58.08	0.0498	3.228	247.42	0	0.01	1/1
DCM (Tihic et al., 2006)	84.93	0.0266	3.338	274.20	–	–	–
ethanol (Gross and Sadowski, 2002)	46.07	0.0517	3.177	198.24	2653.4	0.0324	1/1
nitrogen (Gross and Sadowski, 2001)	28.01	0.0430	3.313	90.96	–	–	–

Wolbach and Sandler(Wolbach and Sandler, 1998) mixing rules were applied for determining the cross-association parameters *ε*^*A*_*i*_*B*_*j*_^/*k*_*B*_ (Eq. (18)) and cross-association volume *κ*^*AiBj*^ (Eq. (19)) in mixtures.

(18) $$\varepsilon^{A_{i}B_{j}}=\frac{1}{2}\cdot \left(\varepsilon^{A_{i}B_{i}}+\varepsilon^{A_{j}B_{j}}\right)$$

(19) κAiBj=κAiBiκAjBjσiσj12σi+σj3

The binary interaction parameter *k*_*ij*_ in Eq. (17) corrects for the deviation of the dispersion energy between unlike segments from the geometric mean of the pure-component parameters and is usually fitted to experimental data of the binary systems. In this work, *k*_*ij*_ was either assumed being constant or to linearly depend on temperature according to (Eq. (20)).(Gross and Sadowski, 2002)

(20) $$k_{\mathrm{ij}}=k_{\mathrm{ij},m}\;T\left(K\right)+k_{\mathrm{ij},b}$$

All binary interaction parameters *k*_*ij*_ used in this work were taken from literature and are summarized in Table 6. The binary *k*_*ij*_ fitted to experimental binary data were used here to predict the phase behavior in the four-component mixtures (API, polymer, solvent + nitrogen).

**Table 6:** Binary interaction parameters *k*_*ij*_ used in this work.

	*k*_*ij,b*_	*k*_*ij,m*_ / K^−1^
NAP/acetone (Luebbert et al., 2018a)	−0.0045	0
NAP/ethanol (Prudic, 2015)	0.0394	−1.35·10^−4^
NAP/DCM (Luebbert et al., 2018a)	0.0113	0
NAP/PVP(Lehmkemper et al., 2017a)	−0.0783	0
NAP/PVPVA64 (Lehmkemper et al., 2017a)	−0.0574	0
RIT/acetone (Dohrn et al., 2021)	0.0245	0
RIT/ethanol (Dohrn et al., 2021)	0.0110	0
RIT/DCM	−0.1498	4.54·10^−4^
RIT/PVPVA64 (Dohrn et al., 2021)	0.0190	0
RIT/PVP (Dohrn et al., 2021)	0.0220	0
PVP/acetone(Luebbert et al., 2018a)	0.0113	0
PVP/ethanol (Luebbert et al., 2018a)	−0.0700	0
PVP/DCM (Luebbert et al., 2018a)	−0.0420	0
PVPVA64/acetone (Dohrn et al., 2020a)	0.0150	−2.7·10^−5^
PVPVA64/ethanol (Dohrn et al., 2020a)	−0.0400	0
PVPVA64/DCM (Dohrn et al., 2020a)	−0.0140	0

## 5 Combined description of macroscopic and microscopic behavior during spray drying

### 5.1 Flowchart for modeling the process design space

**Fig. 4:**
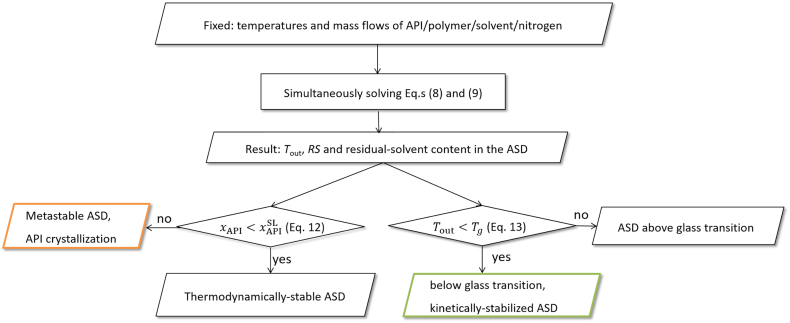
Modeling approach for calculating the process design space. Mass and energy balances were combined with calculating phase equilibrium and glass transition of the ASD/solvent/nitrogen system.

The flowchart for defining the process design space is developed by combining the macroscopic modeling (mass and energy balances) of the spray dryer and the microscopic phase-behavior modeling of the ASD/solvent system as shown in Fig. 4.

The modeling approach for the process design space first considered the macroscopic behavior of the spray dryer for given inlet temperatures *T*_in,N2_ and *T*_in,feed_, mass of nitrogen *m*_N2_ and feed *m*_feed_ and compositions of the liquid feed (*w*_API_, *w*_polymer_ and *w*_solvent_). Solvent vaporization and the resulting temperature were calculated by simultaneously solving Eq.s (8) and (9). As a result, the outlet temperature *T*_out_, *RS*, and residual-solvent content in the ASD at the end of drying were obtained.

After that, the API-crystallization risk of the obtained ASD after the spray-drying process was determined by calculating the temperature-dependent API solubility in the ASD loaded with the residual-solvent content as function of *RS* (Eq. (12)). For that purpose, the API solubility in the ASD loaded with the residual solvent after drying was compared to the API load in the ASD at the same conditions. API loads higher than the API solubility result in metastable ASDs with API-crystallization risk after the spray-drying process. It was further investigated whether the spray-dried ASD was found above or below its glass transition (Eq. (13)). To obtain the process design space for a spray dryer, these calculations were repeated for different inlet drying temperatures and *m*_feed_/*m*_N2_ ratios.

### 5.2 Process design space for spray-drying ASDs

**Fig. 5:**
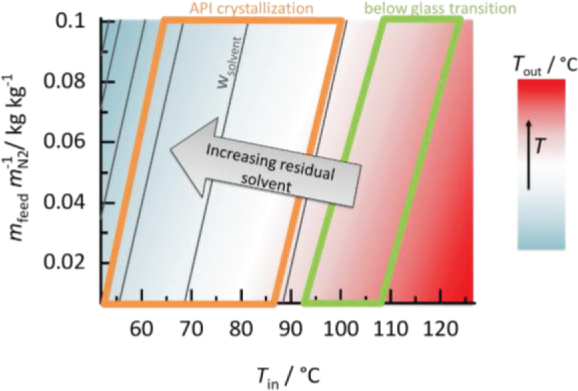
Schematic spray-drying process design space of an ASD. The variable spray-drying parameters are the nitrogen inlet temperature (x-axis) and the relative mass of nitrogen used for the drying process (y-axis). The spray dryer outlet temperatures are indicated by colors. Black lines represent constant residual-solvent contents in the ASD at the end of the drying process. The orange-framed region is the API-supersaturated region in which API crystallization might occur while the region below glass transition is framed green. (For interpretation of the references to colour in this figure legend, the reader is referred to the web version of this article.)

The process design space of ASDs is determined by the macroscopic process conditions as well as the intermolecular interactions between solvent, polymer, and API (Fig. 5).

The spray-dryer process design space comprises the nitrogen inlet temperature (*T*_in,N2_) and the mass ratio between the API/polymer/solvent feed and the nitrogen (*m*_feed_ *m*_N2_^−1^). The initial solvent mass fraction was set to *w*_solvent_ = 0 in the nitrogen and to *w*_solvent_ = 0.97 in the feed (corresponding to 3 wt% total mass fraction of API and polymer in the feed mixture). The inlet temperature of the feed was set to 25 °C. The inlet temperature and solvent *RS* (adjusted by nitrogen as drying gas) are the two properties determining the driving force for solvent drying. High inlet temperatures and a high nitrogen excess result in high solvent drying rates and low residual-solvent contents in the final ASD product. In contrast to that, low inlet temperatures and high solvent *RS* lead to drying products with high residual-solvent contents in the ASD. Drying stops when nitrogen is fully saturated with solvent (*p*_solvent_/*p*_solvent_^LV^ = *RS* = 1) (Eq. (9)). Solvent evaporation leads to a temperature decrease in the spray dryer (evaporative cooling). The outlet temperature of the spray dryer (Eq. (8)) therefore depends on the mass of solvent that evaporated and in turn determines the end of the drying by the temperature-dependent vapor-liquid equilibrium between the solvent-loaded nitrogen and the ASD with the residual solvent content (Eq. (9)) at the spray-dryer outlet.

For determining the ASD-drying process design space, the input parameters nitrogen inlet temperature and feed/nitrogen ratio (*m*_feed_ *m*_N2_^−1^) were not only determined according to their drying performance, but also considering other limitations: While the minimum values of both, inlet temperature and nitrogen mass are limited by the solvent drying performance, the maximum inlet temperature is limited by the product degradation temperature and energy costs.(Newman, 2015) The maximum mass of nitrogen is further limited by the process costs(Dobry Dan et al., 2009) and limits the lower side of the spray-dryer process design space (Fig. 5).

ASD-specific properties like physical stability or residual-solvent content are additional factors to be considered when defining the process design space. Physical changes might occur in the ASD e.g. when exceeding the API solubility (orange-framed region in Fig. 5) potentially leading to API crystallization (Eq. (12)). In addition, temperature as well as residual-solvent content can shift the glass transition (Eq. (13)) of an ASD. Drying conditions should therefore be chosen in a way that the process directly yields powder which does not exceed the glass transition(Hancock et al., 1995; Hancock and Zografi, 1994) (green-framed region in Fig. 5). The following result section investigates the influences of ASD composition, solvent type, and kind of polymer on the process design space considering ASD-quality attributes, such as residual-solvent content, glass transition and possible phase changes during drying.

## 6 Results

### 6.1 *h-X* diagrams

**Fig. 6:**
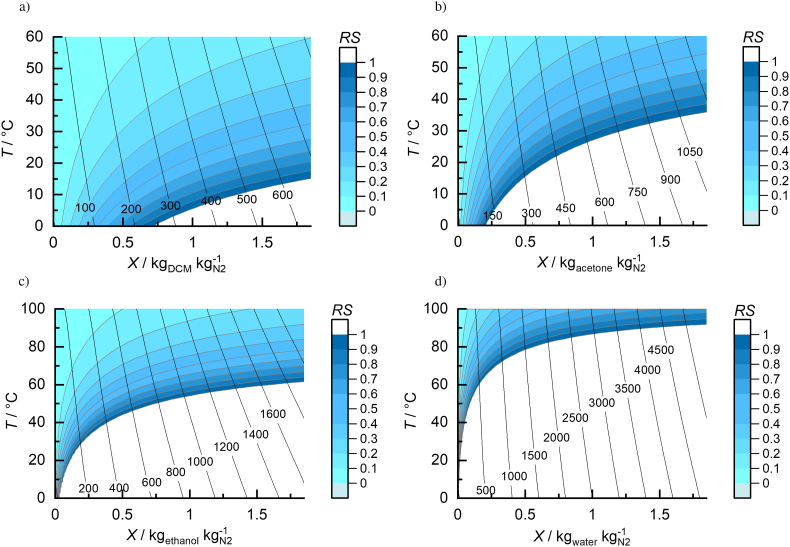
*h-X* diagrams of solvent/nitrogen vapor mixtures at 1.013 bar. The black lines reflect constant *h*_*1+x*_ given in kJ kg^−1^. a) DCM/nitrogen b) acetone/nitrogen c) ethanol/nitrogen, d) water/nitrogen.

*h-X* diagrams for the solvent/nitrogen systems considered in this work are shown in Fig. 6. They reveal the gas-phase properties (*RS* < 1). It should be noted, that for visualization purposes, temperature is given on the y-axis, the solvent load *X* (Eq. (1)) on the x-axis, and constant *h*_*1+X*_ values (Eq. (3)) can be found along lines included in the diagram.

These diagrams for DCM/nitrogen (Fig. 6a), acetone/nitrogen (Fig. 6b), ethanol/nitrogen (Fig. 6c) and water/nitrogen (Fig. 6d) help to graphically compare the different vapor-phase properties at drying-process conditions. Although water was not considered in this study, its diagram is also included for comparison. DCM (*T*^LV^ = 40 °C at 1.013 bar) and acetone (*T*^LV^ = 58 °C at 1.013 bar) were investigated in the temperature range from 0 °C to 60 °C, while *h-X* diagrams for systems containing ethanol and water were calculated in a temperature range from 0 °C to 100 °C at 1.013 bar.

Fig. 6 nicely illustrates a huge solvent influence on the shape of the *h-X* diagrams. This influence arises from the differences in vapor pressures, heat capacities, and vaporization enthalpies of the individual solvents. This results in a solvent-dependent load capacity of nitrogen until *RS* = 1 is reached and drying inevitably stops. The highest solvent load is achievable in DCM systems, followed by acetone and ethanol. In addition to the solvent-dependent load, vapor enthalpies *h*_1+*X*_ remarkably differ for the investigated solvent/nitrogen systems. Similar solvent load leads to the lowest vapor enthalpies for DCM/nitrogen vapor and to highest values for water/nitrogen. This is caused by the fact that evaporating water requires obviously most energy compared to the other solvents whereas the energy demand is lowest in the case of DCM.

*h-X* diagrams are a useful tool for the graphical visualization of the solvent-vapor state and for designing spray-drying processes. These diagrams can be directly calculated with PC-SAFT for any solvent/nitrogen combination. When determining the process design space for spray-drying ASDs, these diagrams give an idea of the solvent influence on the process conditions of the drying processes. However, *h-X* diagrams do not account for non-idealities of the vapor phase neither for the phase behavior of the API/polymer/solvent liquid phase during drying. The latter is considered for when calculating process design spaces in Section 6.2.

### 6.2 Process design spaces

#### 6.2.1 Influence of ASD compositions

##### RIT/PVP ASDs

**Fig. 7:**
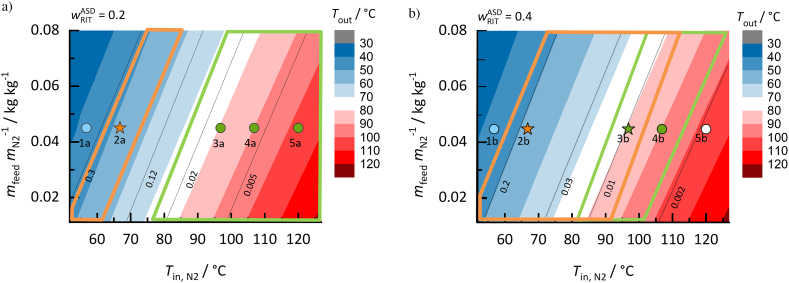
Process design spaces for drying DCM from RIT/PVP ASDs a) RIT load in the solvent-free ASD *w*_RIT_^ASD^ = 0.2 b) RIT load in the solvent-free ASD *w*_RIT_^ASD^ = 0.4. Outlet temperatures decrease from red to blue colored regions; black lines represent the predicted residual-solvent content in the ASD (kg_DCM_/kg_ASD+DCM_). Orange-framed regions are predicted API-supersaturated regions, while green-framed regions are predicted glassy regions. Symbols represent the state at the end of drying for specific process conditions in detail studied in this work. Circles represent process conditions resulting in thermodynamically-stable ASDs, stars represent conditions resulting in ASDs that might crystallize and green-filled symbols represent conditions resulting in ASDs below their glass transition. Conditions resulting in thermodynamically and kinetically unstable ASDs are represented by orange stars and those results in crystal-free solvent/API/polymer mixtures above glass-transition are shown as blue or white circles. (For interpretation of the references to colour in this figure legend, the reader is referred to the web version of this article.)

The process design space for drying RIT/PVP ASDs with two RIT loads of 20 wt% and 40 wt% (*w*_RIT_^ASD^ = 0.2 and *w*_RIT_^ASD^ = 0.4) are shown in Fig. 7a and Fig. 7b, respectively. DCM was chosen as model solvent for the spray-drying process.

When comparing the two process design spaces, the outlet temperatures as well as residual-solvent amounts are not significantly affected by the API load. This is due to the fact that the solvent-sorption behavior is only weakly affected by the different API load. However, the RIT load strongly influences the regions of glass transition (green) and RIT crystallization (orange) inside the process design space. The RIT crystallization region significantly increases when spray drying the ASD with *w*_RIT_^ASD^ = 0.4 (Fig. 7b) compared to the one with the lower load of *w*_RIT_^ASD^ = 0.2 (Fig. 7a). To obtain a thermodynamically-stable ASD outside the API-crystallization region, it is thus suggested to increase the spray-dryer inlet temperature for increasing RIT load. The RIT load further influences the size of the glassy region. For the *w*_RIT_^ASD^ = 0.2 ASD (Fig. 7a), spray-dryer outlet temperatures above 57 °C always result in thermodynamically-stable ASDs and outlet temperatures above 70 °C within the process design space result in ASDs being below glass transition. For *w*_RIT_^ASD^ = 0.4, the glassy region in the process design space is smaller and requires outlet temperatures above 75 °C and below 97 °C. Reaching the glassy state during drying is particularly relevant for spray drying thermodynamically-metastable (supersaturated) ASDs. This can be seen in Fig. 7b, where the crystallization region and the glassy region overlap for certain process conditions. In this regions, RIT-supersaturated but kinetically-stabilized ASDs are obtained after drying. Symbols in Fig. 7a and Fig. 7b denote different final states of RIT/PVP ASDs obtained after drying at different conditions within the process design space. For these five examples, the mass of nitrogen used for the drying process was kept constant (*m*_feed_ *m*_N2_^−1^ = 0.045), while the inlet temperatures of the spray-drying process were increased from 57 °C to 120 °C. The predicted values for outlet temperatures and residual-solvent concentrations in the ASD product are listed in Table 7.

**Table 7:** Spray-drying outlet conditions for the RIT/PVP/DCM process design space shown in Fig. 7.

	*T*_in, N2_ / °C	*m*_feed_*m*_N2_^−1^/ kg kg^−1^	*T*_out_ / °C	*w*_DCM_	*w*_RIT_	*w*_PVP_	ASD state
*w*_RIT_^ASD^= 0.2							
1a	57	0.045	43	0.530	0.094	0.376	thermodynamically-stable ASD above glass transition
2a	67	0.045	52	0.225	0.155	0.620	RIT-supersaturated ASD above glass transition
3a	97	0.045	80	0.012	0.198	0.790	thermodynamically-stable ASD below glass transition
4a	107	0.045	90	0.006	0.199	0.795	thermodynamically-stable ASD below glass transition
5a	120	0.045	104	0.002	0.200	0.798	thermodynamically-stable ASD below glass transition

*w*_RIT_^ASD^ = 0.4							
1b	57	0.045	43	0.540	0.184	0.276	thermodynamically-stable ASD above glass transition
2b	67	0.045	52	0.141	0.344	0.515	RIT-supersaturated ASD above glass transition
3b	97	0.045	80	0.009	0.396	0.595	RIT-supersaturated ASD below glass transition
4b	107	0.045	90	0.005	0.398	0.597	thermodynamically-stable ASD below glass transition
5b	120	0.045	106	0.001	0.399	0.599	thermodynamically-stable ASD above glass transition

Applying a nitrogen inlet temperature *T*_in, N2_ = 57 °C for drying the ASD with a RIT load in the solvent-free ASD *w*_RIT_^ASD^ = 0.2 (state 1a in Fig. 7a) leads to a thermodynamically-stable ASD, but also to an unsuitable-high residual-solvent concentration (*w*_DCM_ = 0.530). Thus, the drying performance would be much too low. By increasing the inlet temperature of the spray dryer to *T*_in, N2_ = 67 °C (2a), the solvent drying is improved (*w*_DCM_ = 0.225) but drying results in RIT-supersaturated ASD now. Inlet temperatures above *T*_in, N2_ = 97 °C lead to thermodynamically-stable ASDs which are above or below (3a, 4a and 5a) the glass transition.

For the RIT/PVP ASD with 40 wt% RIT load (Fig. 7b), low inlet temperatures (1b and 2b) lead to very similar results as for the ASDs with lower RIT load. At *T*_in_ = 97 °C (3b), the resulting ASDs are still located within the RIT-crystallization region but also below glass transition. Thus, the ASD resulting in state 3b is at least kinetically-stabilized. An inlet temperature of *T*_in, N2_ = 107 °C (4b) results in a thermodynamically-stable ASD below glass transition (*T*_out_ = 90 °C and *w*_DCM_ = 0.005). An even further increased drying inlet temperature of *T*_in, N2_ = 120 °C (5b) again results in a stable ASD above glass transition and moreover in the lowest residual-solvent content (*T*_out_ = 106 °C and *w*_DCM_ = 0.001). The ICH-tolerated DCM concentration is 600 ppm (*w*_solvent_ = 0.0006)(ICH Expert Working Group, 2019) and thus, a secondary drying step would be required for all process conditions.

**Fig. 8:**
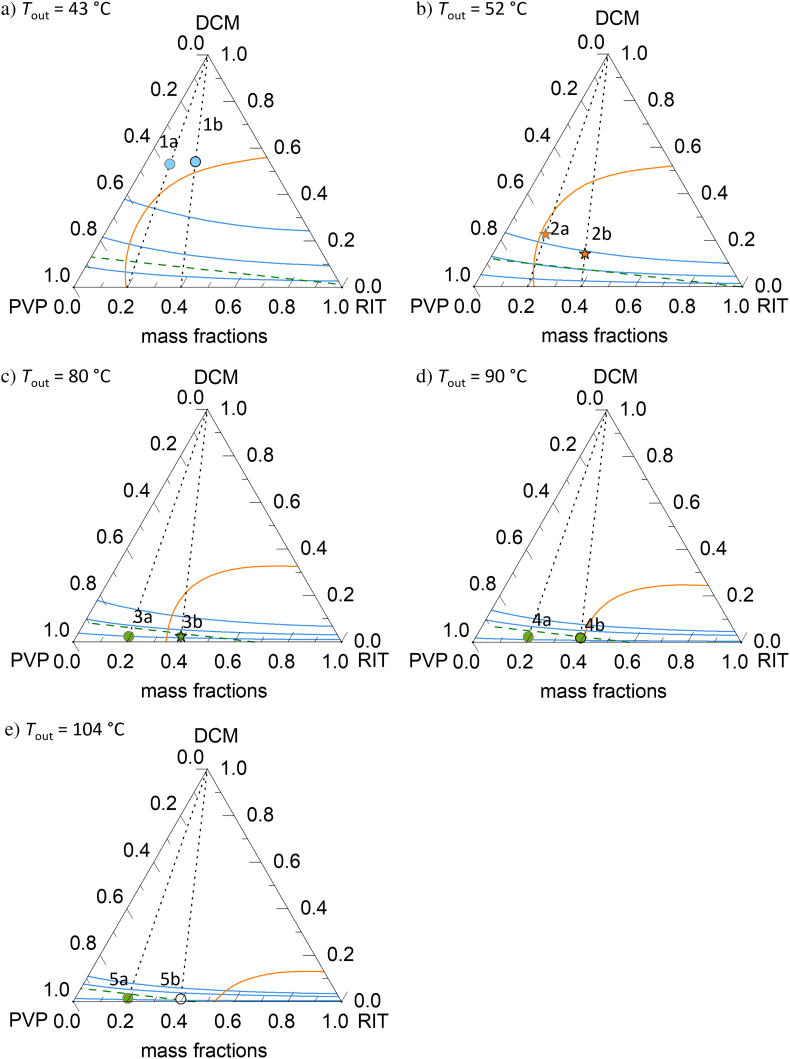
RIT/PVP/DCM ternary phase diagrams at a) *T*_out_ = 43 °C b) *T*_out_ = 52 °C c) *T*_out_ = 80 °C d) *T*_out_ = 90 °C e) *T*_out_ = 104 °C. Orange lines indicate PC-SAFT-predicted solubilities. Blue lines denote constant values of DCM *RS* (0.1; 0.3 and 0.6) also predicted via PC-SAFT. The Gordon-Taylor-predicted glass transitions are green-dashed lines. The drying process is illustrated for two different initial feed compositions with API loads of *w*_RIT_^ASD^ = 0.2 and 0.4 in the solvent-free ASD by black dotted lines and symbols represent the end of drying at conditions shown in Fig. 7. Circles represent process conditions resulting in thermodynamically-stable ASDs, stars represent conditions resulting in ASDs that might crystallize and green-filled symbols represent conditions resulting in ASDs below their glass transition. (For interpretation of the references to colour in this figure legend, the reader is referred to the web version of this article.)

Fig. 8 shows the API/polymer/solvent phase diagrams obtained for the predicted outlet temperatures for the example process conditions discussed above (Fig. 7). Drying runs along the lines from the solvent-rich corner at the top of the triangle diagrams to the ends of drying (1a-5a for *w*_RIT_^ASD^ = 0.2; 1b-5b for *w*_RIT_^ASD^ = 0.4) at the solvent-free bottom sides of the triangles. The residual-solvent content in the ASDs decreases from points 1 to points 5. *T*_out_ for condition 5b is 106 °C (5a is 104 °C), however, the impact of the small temperature difference on the phase diagram is negligibly small. A homogeneous, thermodynamically-stable ASD is obtained when the drying ends are located left of the solubility line (circles). ASDs found within the RIT-crystallization region in Fig. 7 are located right of the solubility line in the ternary phase diagrams. Such ASDs are RIT-supersaturated but might be kinetically-stabilized when stored below glass transition (e.g. 3b). In general, ASDs above glass transition are obtained when drying ends above the glass-transition line and kinetically-stabilized ASDs are located below the glass transition lines.

#### 6.2.2 Influence of solvents

##### DCM vs. ethanol for NAP/PVP ASDs

**Fig. 9:**
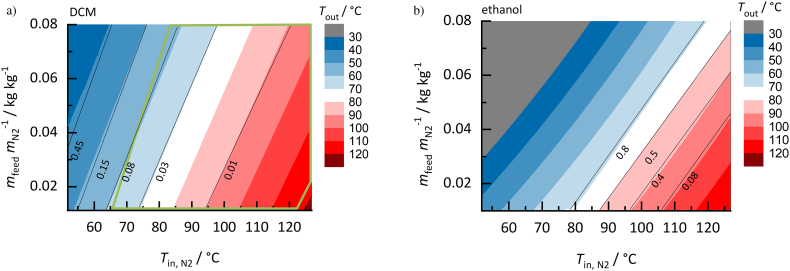
Process design spaces for drying a) DCM and b) ethanol from NAP/PVP ASD (NAP load in the solvent-free ASD *w*_NAP_^ASD^ = 0.2). Calculated outlet temperatures decrease from red to blue colored regions; black lines represent the predicted residual-solvent content (kg_solvent_/kg_ASD+solvent_) in the ASD indicated by numbers. Green-framed region is the predicted glassy region. (For interpretation of the references to colour in this figure legend, the reader is referred to the web version of this article.)

Fig. 9 compares the process design space for NAP/PVP ASDs (NAP load in the solvent-free ASD in both cases *w*_NAP_^ASD^ = 0.2) spray dried with DCM (Fig. 9a) or ethanol (Fig. 9b).

As to be seen, the outlet temperatures *T*_out_ and the residual-solvent concentrations are strongly affected by the solvent. The different solvent evaporation temperatures (*T*_ethanol_^LV^ = 78 °C; *T*_DCM_^LV^ = 40 °C at 1.013 bar) and enthalpies of evaporation as well as the different solvent volatilities in the ternary API/polymer/solvent mixtures result in very different process design spaces. The same inlet conditions for DCM (Fig. 9a) and ethanol (Fig. 9b) lead to much lower outlet temperatures (*T*_out_) and significantly higher residual-solvent concentrations for ethanol than for DCM. Thus, higher inlet temperatures and higher mass of nitrogen are required to evaporate ethanol from the ASD. Almost no ethanol evaporates (*w*_ethanol_ > 0.9) from the liquid feed at spray-drying outlet temperatures below *T*_out_ = 65 °C. This is in accordance with the solvent/nitrogen *h-X* diagrams (Fig. 6a and Fig. 6c), indicating a less-efficient drying performance for ethanol/nitrogen systems compared to DCM/nitrogen systems due to the higher solvent load of DCM in the vapor and less energy required for DCM evaporation compared to that of ethanol.

Moreover, when using ethanol, none of the obtained ASDs within the presented diagram is below glass transition (Fig. 9b) after drying, while using DCM can lead to glassy ASDs depending on the process conditions for outlet temperatures between 60 and 118 °C (Fig. 9a). With both solvents, thermodynamically-stable ASDs are obtained after drying, since no NAP crystallization was predicted for any process conditions within the considered parameter ranges. When comparing the solvents, DCM seems to be the more-appropriate solvent for spray drying the investigated NAP/PVP ASD due to the moderate drying conditions required.

##### DCM vs. acetone for RIT/PVPVA64 ASDs

**Fig. 10:**
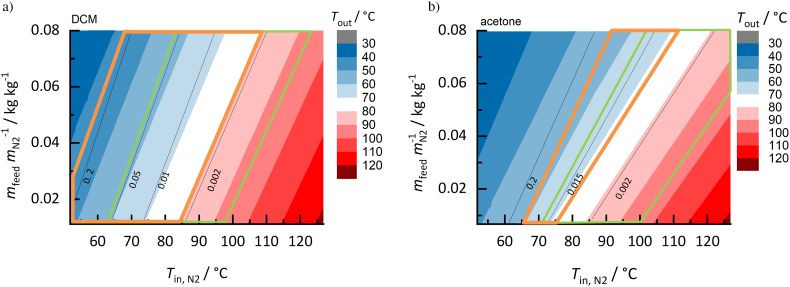
Process design spaces for drying a) DCM and b) acetone from RIT/PVPVA64 ASD (RIT load in the solvent-free ASD *w*_RIT_^ASD^ = 0.2). Outlet temperatures decrease from red to blue colored region; black lines represent the predicted residual-solvent content in the ASD (kg_solvent_/kg_ASD+solvent_). Orange-framed regions are RIT-supersaturated regions and green-framed regions are the predicted glassy regions. (For interpretation of the references to colour in this figure legend, the reader is referred to the web version of this article.)

As a second example for the solvent impact, spray drying of RIT/PVPVA64 ASDs (RIT load in the solvent-free ASD *w*_RIT_^ASD^ = 0.2) with DCM (Fig. 10a) or acetone (Fig. 10b) was investigated.

Although acetone has a significantly higher boiling temperature than DCM (*T*_acetone_^LV^ = 56 °C; *T*_DCM_^LV^ = 40 °C at 1.013 bar), the solvent-drying performance for RIT/PVPVA64 ASDs is quite similar for the two solvents. The process design spaces differ slightly in their outlet temperatures and residual-solvent amounts. However, the solvent effect on the size of the crystallization region and glass transition is tremendous: a significantly smaller RIT crystallization region and smaller overlap with the glass-transition region is observed when using acetone instead of DCM. Since physical changes might occur in the ASD when exceeding the API solubility, this orange-framed crystallization region in the design space should be avoided. Further, drying conditions in the green-framed regions are preferred as they yield product, which does not exceed its glass transition. Acetone therefore seems to be the more-appropriate solvent compared to DCM for the investigated RIT/PVPVA64 ASD, since no RIT crystallization can occur in the final ASDs for outlet temperatures above 71 °C and glassy ASDs are obtained in a large temperature region up to an outlet temperature of 96 °C. Using DCM as solvent, likewise leads to a large region below glass transition, however most of the glassy region is covered by the RIT crystallization region. DCM thus reveals a higher risk of RIT crystallization during/after drying compared to acetone.

This example demonstrates that the solvent with the highest evaporation propensity is not necessarily the most-appropriate one for a spray-drying process. For RIT/PVPVA64 ASDs, acetone should be the preferred as it allows for obtaining thermodynamically-stable ASDs below glass transition at moderate spray-drying conditions. In contrast, DCM reveals a higher risk of RIT crystallization during/after drying. It can be deduced from this example that the solvent may have a dramatic influence on the process design space and that different solvents can result in completely different process design spaces and crystallization behaviors, although the final ASD product is solvent-free in both cases.

#### 6.2.3 Influence of polymers

##### PVP vs. PVPVA64 for NAP ASDs

**Fig. 11:**
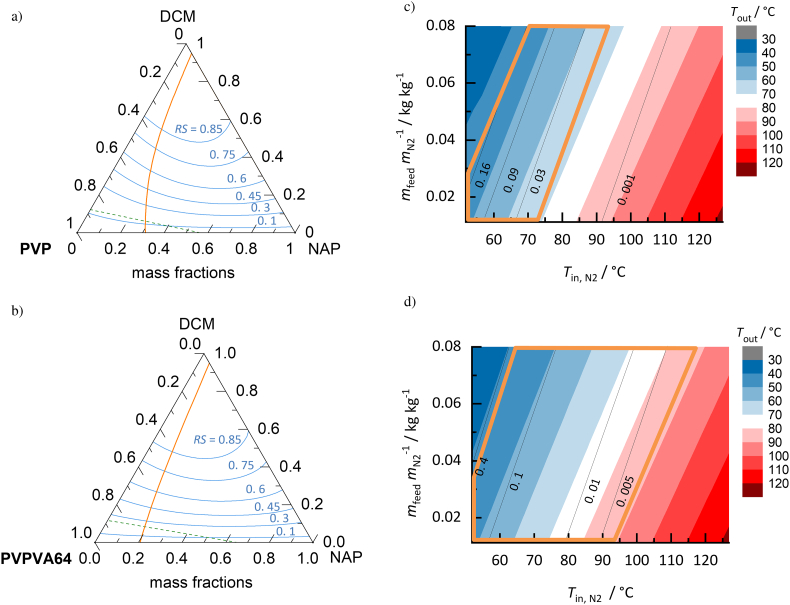
Ternary phase diagrams (*T* = 25 °C) and process design spaces for drying DCM from a) NAP/PVP and b) NAP/PVPVA64 ASDs (NAP load in the solvent-free ASD *w*_NAP_^ASD^ = 0.4). Orange lines are solubility lines, green dashed lines are the glass-transition lines and blue lines are *RS* lines from 0.1 to 0.85 in the ternary phase diagrams. In the process design spaces of c) NAP/PVP/DCM and d) NAP/PVPVA64/DCM, the outlet temperature decreases from red-colored to blue-colored regions; while black lines represent the predicted residual-solvent content in the ASDs (kg_DCM_/kg_ASD+DCM_). Orange-framed regions are NAP-supersaturated regions. (For interpretation of the references to colour in this figure legend, the reader is referred to the web version of this article.)

The influence of the polymer on the process design space was studied for the polymers PVP (Fig. 11a and Fig. 11c) and PVPVA64 (Fig. 11d and Fig. 11e) for NAP ASDs dried from DCM solutions.

PVP leads to an increased NAP solubility (Fig. 11a) compared to the use of PVPVA64 (Fig. 11b). The glass transitions in the ternary systems are only slightly different for the two polymers (*T*_*g,*PVPVA64_ = 111 °C(Prudic et al., 2014b); *T*_*g,*PVP_ = 173 °C(Dohrn et al., 2020a)). However, the solvent-sorption behavior is significantly influenced by the choice of the polymer, which can be seen when comparing the *RS* lines in Fig. 11a and Fig. 11b. At the same temperature and the same *RS*, the PVP-containing system absorbs more DCM than the PVPVA64 system. This effect decreases with increasing API content in the mixture. The influence of the polymer on the solvent-sorption behavior also becomes apparent by the slightly different outlet temperatures and residual-solvent amounts in the calculated process design spaces (Fig. 11c and Fig. 11d) for NAP ASDs with *w*_NAP_^ASD^ = 0.4.

Moreover, the polymer strongly influences the size of the crystallization region and the glass transition region in the process design space. The NAP solubility in the PVP/DCM mixture is higher compared to the one in the PVPVA64/DCM mixture (compare Fig. 11a and Fig. 11b). This leads to a NAP-supersaturated region in the process design space of the PVPVA64/DCM mixture(Dohrn et al., 2021) whereas a smaller NAP-supersaturated region was found for the PVP/DCM mixture at outlet temperatures below 70 °C. All ASDs obtained within the process design spaces were predicted to be above glass transition; therefore, no kinetic stabilization is expected for NAP/PVPVA64 ASDs within the crystallization region. This comparison illustrates that the polymer may strongly affect the residual solvent content in the ASDs. For example, to obtain thermodynamically-stable ASDs, the spray-dryer inlet temperatures need to be chosen higher when using PVPVA64 instead of PVP for NAP containing ASDs spray-dried with DCM.

## 7 Conclusion

This work predicted design spaces for ASD spray-drying process encompassing solvent-drying performance, spray-dryer outlet temperatures, residual-solvent content in the final ASDs, risk of API crystallization in the ASD, and regions of glass transition. It was shown that different drying inlet parameters have a decisive influence on the stability of the obtained ASD after drying. By investigation of *h-X* diagrams, it became visually clear that solvent drying differs due to the different solvent-load capacities of the drying gas, nitrogen. Due to the highest volatility of DCM compared to acetone or ethanol (DCM has the lowest boiling temperature and the lowest evaporation enthalpy), the highest load *X* was predicted to be achievable with DCM as solvent for the drying process. However, *h-X* diagrams are only of limited use in designing the ASD spray-drying process, since they consider only the nitrogen/solvent systems and do not account for non-ideality in the vapor phase nor for the API and the polymer in the liquid phase. The influence of solvents, polymers, and API load on the drying performance and on ASD stability was therefore accounted for using PC-SAFT when predicting the process design spaces in this work. It was shown that different drying conditions decisively influence the drying performance and the ASD stability, resulting in different outlet temperatures and residual-solvent contents in the ASD. The influence of the API load on the spray-drying outlet temperature and residual-solvent content was investigated for RIT/PVP ASDs with DCM as solvent and was found to be small. However, by adjusting the nitrogen inlet temperature while keeping the kind of solvent and API/polymer/solvent feed constant, it is possible to obtain ASDs above or below glass transition, metastable API-supersaturated or thermodynamically-stable ASDs. To avoid API crystallization and to generate ASDs below the glass transition, the required inlet temperature (at constant feed rate) significantly depends on the ASD composition. The solvent influence was predicted for NAP/PVP and RIT/PVPVA64 ASDs spray dried using the solvents DCM, ethanol, and acetone. It was found that the solvent-drying performance strongly differs for the same ASD, while additionally the solvent strongly influences the potential risk of API crystallization as well as the glass transition at the end of the drying process. Thermodynamically-stable NAP/PVP ASDs were predicted to be obtained in the whole process design spaces using DCM or ethanol. Nevertheless, due to the more-moderate drying conditions required for a certain residual-solvent content, DCM seems to be the more appropriate solvent compared to ethanol for these ASDs. In case of RIT/PVPVA64 ASDs, it was found that acetone seems to be the more appropriate solvent compared to DCM. Although the drying performance of acetone was predicted being slightly poorer compared to DCM, the risk of RIT crystallization can be reduced using acetone. The polymer influence on the drying performance was investigated for NAP/PVPVA64 and NAP/PVP ASDs spray dried with DCM. It was found that the polymer mainly influences the NAP solubility, while in these systems the solvent-sorption behavior and glass transition was only slightly affected by the polymer. Due to the lower NAP solubility in PVPVA64 compared to PVP, the spray-dryer inlet temperature needs to be higher when using PVPVA64 for obtaining a thermodynamically-stable ASD.

This work thus presents an approach for predicting process design spaces for spray-drying ASDs allowing for the best-achievable product quality without carrying out spray-drying experiments. It could be shown that the process design space of ASDs does not only depend on mass and energy balances, but also on the intermolecular interactions in the API/polymer/solvent system. Knowing the thermodynamic phase behavior comprising API solubilities, solvent sorption and glass transitions combined with spray-dryer mass and energy balances enable identification of spray-drying conditions at which ASDs do not crystallize, contain low amounts of residual solvent, and/or lie below glass transition. The approach therefore is a useful tool for choosing appropriate solvent candidates and process conditions for the ASD spray drying with minimal experimental effort. These process conditions also affect other product properties, like size and shape of the particles and therewith also their dissolution rate. The proposed approach can therefore also be used to support a Design of Experiments in process development.

## Nomenclature

*a*: Helmholtz energy
A_i_,B_i_: association sites A and B of molecule
API: active pharmaceutical ingredient
ASD: amorphous solid dispersion
*Δc*_*p*, API_^SL^: difference in solid and liquid heat capacity
*c*_*p*_: heat capacity
DCM: dichloromethane
DoE: Design of Experiment
*h*_*1+X*_: enthalpy
∆*h*_solvent_^LV^: solvent evaporation enthalpy
Δ*h*_API_^SL^: API melting enthalpy
ICH: International Council for Harmonisation
*K*: Gordon-Taylor interaction parameter
*k*_*B*_: Boltzmann constant
*k*_*ij*_: binary interaction parameter
*m*: mass
m_seg_: segment number
*M*_*w*_: molecular weight
NAP: naproxen
*N*^*assoc*^: number of association sites
N_2_: nitrogen
*p*: pressure
*p*^LV^: vapor pressure
PC-SAFT: Perturbed-Chain Statistical Associating Fluid Theory
PVP: poly (vinylpyrrolidone)
PVPVA64: poly (vinylpyrrolidone-*co*-vinylacetate)
*R*: universal gas constant
*RH*: relative humidity
RIT: ritonavir
*RS*: relative saturation
SLE: solid liquid equilibrium
*T*: temperature
*T*^*c*^: critical temperature
T_g_: glass transition temperature
*T*^LV^: boiling temperature
*T*_ref_: reference temperature
*T*_API_^SL^: API melting temperature
*u*: dispersion-energy
VLE: vapor liquid equilibrium
*w*: mass fraction
*x*: mole fraction
*X*: load

### Greek characters

*γ*: activity coefficient
*ε*_*AiBi*_*/ k*_*B*_: association-energy parameter
*ρ*: density
*κ*_*AiBi*_: association-volume parameter
*σ*: segment diameter

### Subscripts

i, j: component
int: intersection

### Superscripts

assoc: association
disp: dispersion
hc: hard-chain
L: liquid
res: residual
S: solid
V: vapor

## Author contributions

The manuscript was written through contributions of all authors. All authors have given approval to the final version of the manuscript.

## Acknowledgements and disclosure

This study was funded by 10.13039/100006483AbbVie. AbbVie participated in the study design, research, data collection, analysis, and interpretation of data, as well as writing, reviewing, and approving the publication. S.K., M.D., and K.L. are AbbVie employees and may own AbbVie stock/options. G.S. is professor, C.L. is postdoc, P.R is student and S.D. is PhD student at the Department of Biochemical and Chemical Engineering at TU Dortmund University and they have no conflict of interest to report.

## Declaration of Competing Interest

None.

## Appendix A Supplementary data

Supplementary material Image 1
