# Supplementary material for: Predicting process design spaces for spray drying amorphous solid dispersions
Source: Int J Pharm X. 2021 Feb 25;3:100072. doi: 10.1016/j.ijpx.2021.100072 (PMC8027772; doi:10.1016/j.ijpx.2021.100072)
Supplement: Supplementary file 1 — Supplementary material [file mmc1.docx]

Supporting Information

Predicting process design spaces for spray drying amorphous solid dispersions

Stefanie Dohrn^1^, Pranay Rawal^1^, Christian Luebbert^1^, Kristin Lehmkemper^2^, Samuel O. Kyeremateng^2*^, Matthias Degenhardt^2^ and Gabriele Sadowski^1*^

^1^ TU Dortmund University, Department of Biochemical and Chemical Engineering, Laboratory of Thermodynamics, Emil-Figge-Str. 70, D-44227 Dortmund, Germany.

^2^ AbbVie Deutschland GmbH & Co. KG, R&D, Knollstraße, D-67061 Ludwigshafen am Rhein, Germany.


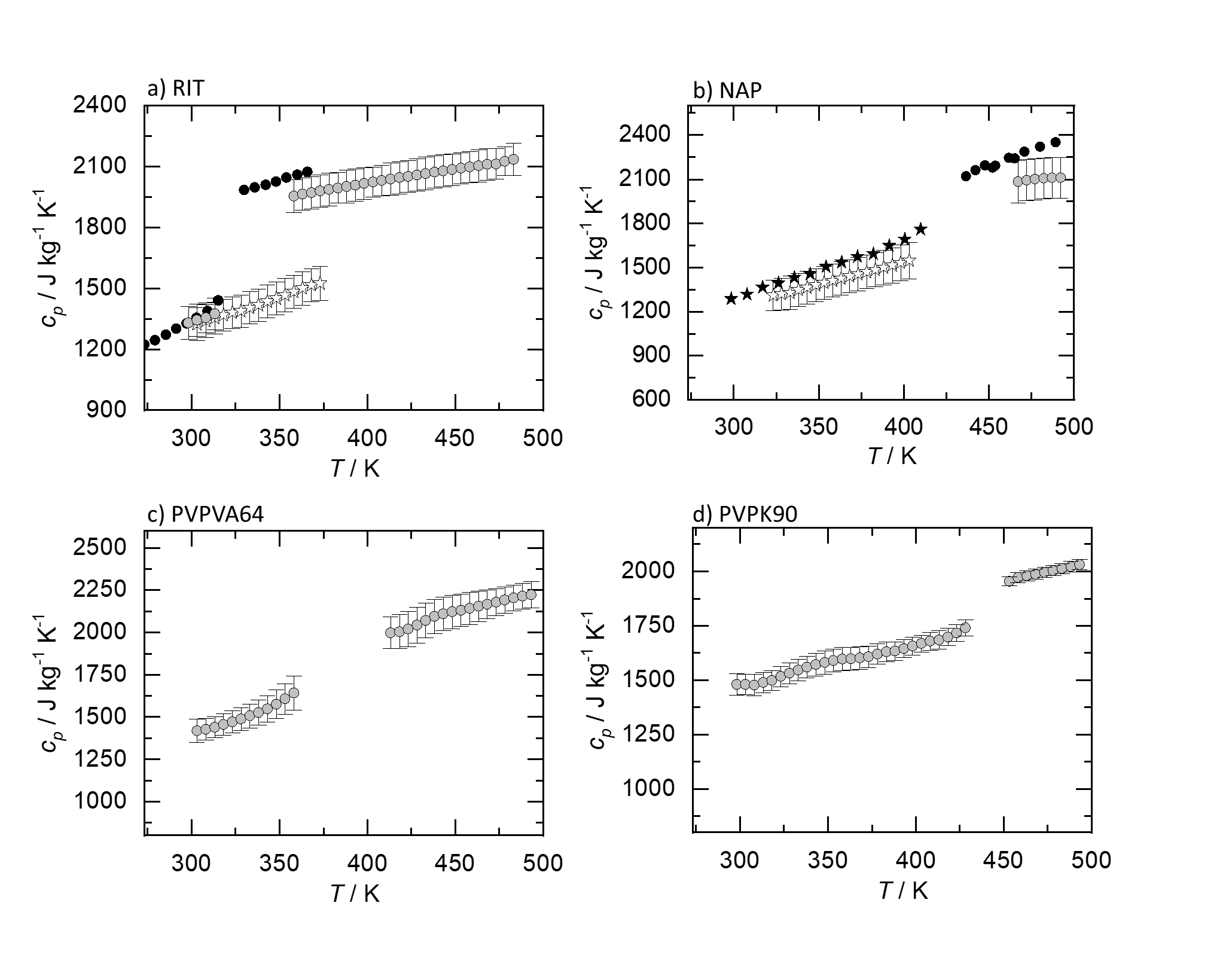


Figure S1: Heat capacity as function of temperature for a) RIT b) NAP c) PVPVA64 and d) PVPK90. Symbols are experimental data points, whereby circles represent amorphous samples, stars represent crystalline samples and black filled symbols are data points from literature (RIT: Zhou et al.^1^) (NAP: Buchholz et al.^2^). The error bars show the standard deviation from three measurements.

Table S1: Heat capacity as function of temperature for RIT, NAP, PVPK90, PVPVA64

| ritonavir (RIT) | | naproxen (NAP) | | PVPK90 | | PVPVA64 | |
| --- | --- | --- | --- | --- | --- | --- | --- |
| *T* / K | *c_p_* / J kg^-1^ K^-1^ | *T* / K | *c_p_* / J kg^-1^ K^-1^ | *T* / K | *c_p_* / J kg^-1^ K^-1^ | *T* / K | *c_p_* / J kg^-1^ K^-1^ |
| crystalline | | crystalline | | amorphous, below *T_g_* | | amorphous, below *T_g_* | |
| 303.15  308.15  313.15  318.15  323.15  328.15  333.15  338.15  343.15  348.15  353.15  358.15  363.15  368.15  373.15 | 1324  1340  1354  1368  1380  1388  1403  1418  1435  1451  1467  1499  1520  1536  1564 | 323.15  328.15  333.15  338.15  343.15  348.15  353.15  358.15  363.15  368.15  373.15  378.15  383.15  388.15  393.15  398.15  403.15 | 1310  1319  1329  1347  1363  1380  1395  1411  1426  1440  1454  1469  1484  1499  1515  1531  1546 | 298.15  303.15  308.15  313.15  318.15  323.15  328.15  333.15  338.15  343.15  348.15  353.15  358.15  363.15  368.15  373.15  378.15  383.15  388.15  393.15  398.15  403.15  408.15  413.15  418.15  423.15  428.15 | 1480  1481  1477  1489  1499  1517  1532  1546  1560  1571  1581  1590  1597  1598  1602  1608  1620  1630  1634  1645  1656  1669  1679  1685  1698  1717  1740 | 303.15  308.15  313.15  318.15  323.15  328.15  333.15  338.15  343.15  348.15  353.15  358.15 | 1418  1426  1439  1456  1471  1488  1506  1526  1548  1575  1607  1642 |
| amorphous, below *T_g_* | | amorphous, above *T_g_* | | amorphous, above *T_g_* | | amorphous, above *T_g_* | |
| 298.15  303.15  308.15  313.15 | 1330  1344  1355  1375 | 467.15  472.15  477.15  482.15  487.15  492.15 | 2083  2094  2101  2106  2110  2110 | 453.15  458.15  463.15  468.15  473.15  478.15  483.15  488.15  493.15 | 1954  1971  1980  1987  1995  2004  2013  2023  2031 | 408.15  413.15  418.15  423.15  428.15  433.15  438.15  443.15  448.15  453.15  458.15  463.15  468.15  473.15  478.15  483.15  488.15  493.15 | 1997  2004  2020  2043  2071  2094  2110  2121  2130  2142  2154  2166  2179  2191  2203  2214  2223  2231 |
| amorphous, above *T_g_* | |  |  |  |  |  |  |
| 358.15  363.15  368.15  373.15  378.15  383.15  388.15  393.15  398.15  403.15  408.15  413.15  418.15  423.15  428.15  433.15  438.15  443.15  448.15  453.15  458.15  463.15  468.15  473.15  478.15  483.15 | 1955  1965  1972  1980  1987  1995  2002  2009  2017  2024  2031  2038  2045  2052  2059  2066  2073  2079  2085  2092  2098  2104  2111  2113  2126  2135 |  |  |  |  |  |  |

References:

(1) Zhou, D.; Zhang, G. G.; Law, D.; Grant, D. J.; Schmitt, E. A. Physical stability of amorphous pharmaceuticals: Importance of configurational thermodynamic quantities and molecular mobility. *J. Pharm. Sci.* **2002**, *91*, 1863–1872.

(2) Buchholz, H.; Emel'yanenko, V.; Lorenz, H.; Verevkin, S. P. An Examination of the Phase Transition Thermodynamics of (S)- and (RS)-Naproxen as a Basis for the Design of Enantioselective Crystallization Processes. *J. Pharm. Sci.* **2016**, *105*, 1676–1683.
